# Un cas rare de luxation astragalo-scapho-calcanéenne interne

**DOI:** 10.11604/pamj.2018.31.91.16874

**Published:** 2018-10-05

**Authors:** Soufiane Aharram, Abdelhafid Derfoufi, Abdessamad Kharraji, Jawad Amghar, Mohammed Benhamou, Abdelkarim Daoudi, Omar Agoumi

**Affiliations:** 1 Service de Traumatologie-Orthopédie, CHU Mohammed VI, Oujda, Maroc

**Keywords:** Accident de sport, jeune patient, luxation sous talienne, Sport accident, young patient, subtalar dislocation

## Abstract

Nous rapportons un cas d'un jeune patient ayant présenté à la suite d'un accident de sport une luxation astragalo-scapho-calcanéenne interne où il a bénéficié d'un traitement orthopédique avec un bon résultat clinique et radiologique.

## Abstract (en)

We here report the case of a young patient presenting with sports-related internal astragalo-scapho-calcaneal dislocation. The patient underwent orthopedic treatment with good clinical and radiological outcome.

## Introduction

La luxation sous talienne est une lésion très rare, représente 1% de toutes les luxations en traumatologie selon certains auteurs [1]. La luxation astragalo-scapho-calcanéenne interne est une perte de rapports anatomiques entre l'astragale, le calcanéum et le scaphoïde, mais la congruence tibio-péronéo-astragalienne est maintenue. Le diagnostic est clinique confirmé par les radiographies du pied et de la cheville. Parfois le recours au scanner est nécessaire à la recherche des lésions ostéo-cartilagineux associées. La réduction doit être réalisée en urgence sous anesthésie. Si la réduction orthopédique s'avère impossible, le traitement chirurgical permet d'obtenir une réduction anatomique en levant les obstacles et en réalisant une ostéosynthèse des fractures intra-articulaires associées. Le pronostic de cette lésion est meilleur si le traitement est rapide et approprié.

## Patient et observation

**Figure 1:**
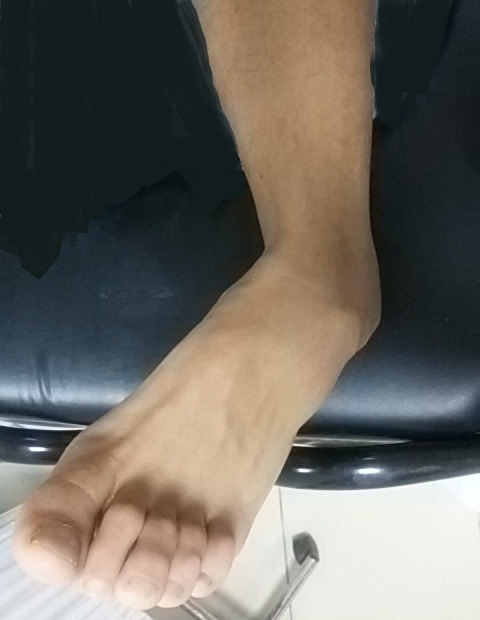
Aspect clinique de la cheville à l’admission du patient à l’urgence

**Figure 2:**
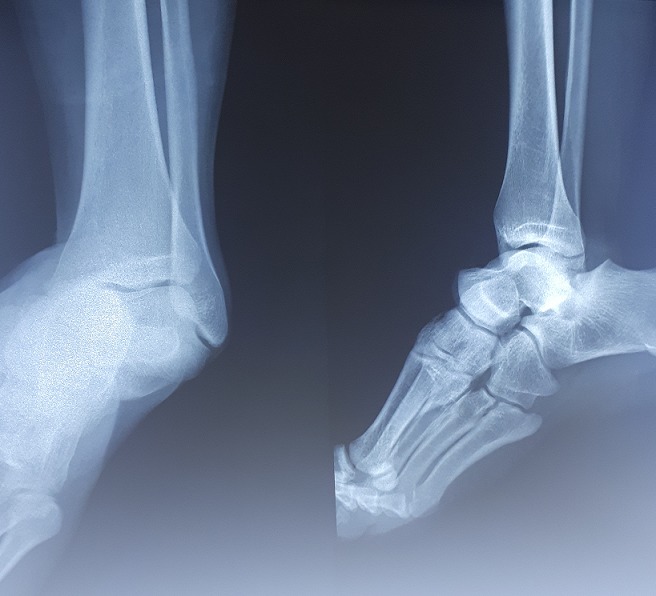
Radiographie standard du pied et de la cheville de face et de profil confirme la luxation sous talienne interne

**Figure 3:**
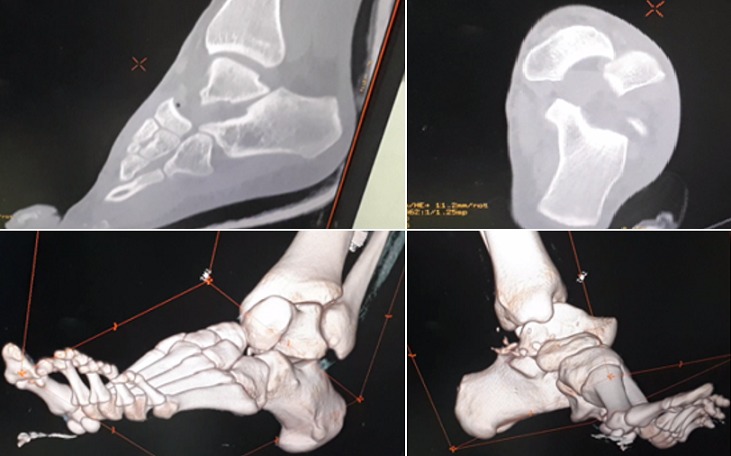
Scanner du pied et de la cheville apprécié l’absence des lésions ostéo-cartilagineux

**Figure 4:**
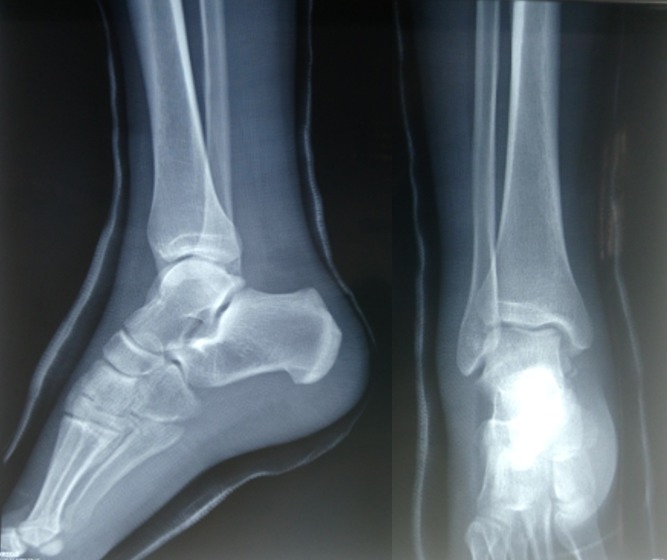
Radiographie standard de contrôle après réduction de la luxation

**Figure 5:**
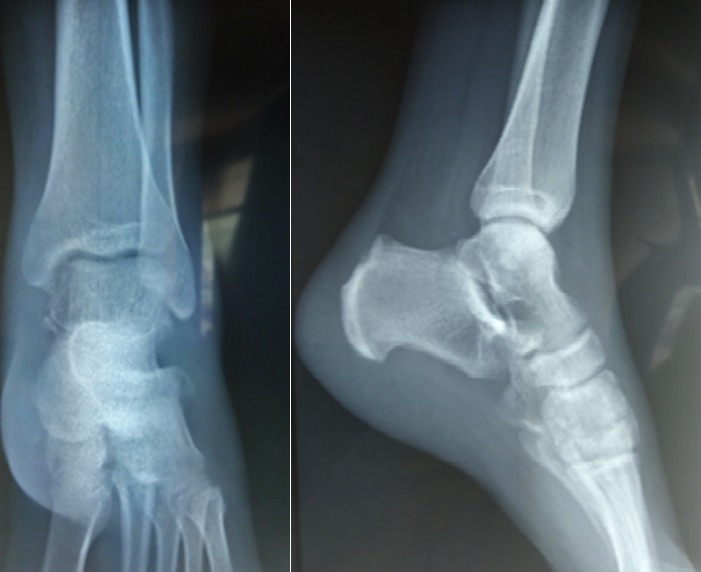
Radiographie après l’ablation du plâtre à 6 semaines

Il d'un patient de 25ans, sans antécédents pathologiques notables, footballeur professionnel, a été victime d'un accident de sport (lors d'un match de football), avec pointe de réception en inversion et équinisme sur le pied gauche, le patient admis à l'urgence pour prise en charge. L'étude clinique avait montré une douleur avec impotence fonctionnelle totale du membre, une déformation de la région médio-tarsienne: le talon est déplacé en interne par rapport à la jambe, le pied étant en inversion, raccourcissement du bord médial du pied et une tension cutanée, avec œdème de la cheville. Il n'y avait pas de souffrance cutanée, ni de lésion vasculonerveuse (Figure 1). Un bilan radiologique objectivait une luxation astragalo-scapho-calcanéenne interne sans fracture associée (Figure 2). Le scanner du pied et de la cheville a confirmé ces lésions (Figure 3). La réduction orthopédique a été réalisée en urgence par dans l'heure suivant le traumatisme au bloc opératoire sous anesthésie générale par manœuvres externes. Après la réduction, l'articulation sous-talienne étant stable à l'examen clinique et le contrôle radiologique avait montré une bonne congruence articulaire pour les articulations, sous-talienne et talo-naviculaire (Figure 4). La cheville a été immobilisée dans une botte plâtrée et maintenue pendant 6 semaines sans autorisation de l'appui, ensuite une rééducation fonctionnelle a été entreprise (Figure 5). Le patient a repris ses activités sportives à 4 mois après le traumatisme. Un recul de 6mois, le résultat fonctionnel était bon.

## Discussion

La luxation astragalo-scapho-calcanéenne est l'un des traumatismes le plus rare de la cheville, la variété interne est la plus fréquente [2]. Le mécanisme des luxations sous astragaliennes interne est discutable avec les auteurs, pour Baumgartner Huguier [3] le faisceau péronéo-calcanéenne du ligament latéral externes déchire successivement, puis le ligament en haie, puis la poussée du pied vers l'intérieur se poursuivant, le ligament astragalo-scaphoîdien finit par se rompre. Giraud et Rachou [4], affirment que la haie interosseuse est trop résistante pour se rompre, et qu'elle s'arrache plutôt de son insertion inférieure. Watson-Jones [5] considère la luxation sous-astragalienne comme le deuxième stade des accidents par inversion du pied (luxation double), le premier stade étant la luxation de la cheville (luxation simple), le troisième étant l'énucléation de l'astragale (luxation triple). Enfin, dans cet éventail de théories élaborées. Nous retiendrons les conclusions de l'étude expérimentale réalisée par Allieu [6] et son équipe qui ont précisé le mécanisme de la luxation sous-astragalienne interne ou luxation astragalo-scapho-calcanéenne: cette étude est basée sur des arguments cliniques, anatomiques, expérimentaux et biomécaniques, qui ont permis d'obtenir les déductions suivants: le sujet subit un traumatisme sous le pied qui est en position fragilisée à savoir INVERSION et EQUINISME (et non pas à angle droit ou fléchi sur la jambe comme le pensent certains auteurs: Baumgartner et Huguier, queriu. La luxation astragalo-scapho-calcanéenne correspond à une luxation totale des articulations sou-astragaliennes antérieure et postérieure, et s'accompagne d'importantes lésions ligamentaires. Il se produit une luxation astragalo-scaphoidienne interne avec lésion du ligament astragalo-scaphoîdien dorsal, la tête astragalienne déchire ensuite le ligament frondiforme (dédoublement du ligament annulaire antérieur du tarse). Et la pression continuant de s'exercer, le faisceau péronéo-calcanéenne du ligament latéral externe finit par se rompre. Cette luxation survient à la suite d'un traumatisme à haute énergie. Elle intéresse l'adulte jeune de sexe masculin dont les circonstances de survenue sont multiples à savoir: les accidents de sport, les accidents de la voie publique, les chutes d'un lieu élevé etc…

Le diagnostic est en général facile devant la déformation évidente de la cheville, le pied fixé en inversion. Des clichés radiographiques de le cheville de face et de profil posent le diagnostic en montrant sur le cliché de face : le calcanéum et l'axe du pied sont déplacés en dedans, l'astragale reste enclavé dans la mortaise et donc sa partie externe repose dans le vide, sur le cliché de profil : l'interligne de l'articulation sous-astragalienne est effacée en raison du chevauchement de l'astragale et du calcanéum, la surface scaphoidienne est déshabitée. Le scanner permet de confirmer le diagnostic et d'apprécier le degré des lésions ostéo-cartilagineux associées [7]. Le traitement consiste à une réduction en urgence sous anesthésie générale en utilisant la manœuvre d'arrache botte avec une contention supplémentaire post réductionnelle pour une durée moyenne de 45 jours [8, 9]. Les méthodes décrites par les auteurs classiques (Boehler [10]) restent valables et nous rappellerons les points essentiels d'une réduction aisée: malade en décubitus dorsal, genou fléchi à 90° pour relâcher le triceps et une main est placée sur la région antérosupérieure de la tibiotarsienne assure le maintien du membre inférieur, l'autre main empaume et tire en avant le pied en flexion plantaire comme pour arracher une botte. Malgaigne [11] recommande d'exercer une impulsion sur tête de l'astragale pour le guider vers la sphère articulaire. Dans les cas difficiles, on peut s'aider d'une broche transcalcanéenne, en particulier s'il existe une fracture du col; cette broche permet une traction dans l'axe de la jambe, puis en bas et en arrière afin de réduire la luxation antérieure; la mise en flexion plantaire du pied réduit la fracture en alignant le col sur le corps en position d'équilibre. Parfois, la réduction peut être instable et impossible. Cette stabilité doit être jugée cliniquement et vérifiée radiologiquement. En effet, les radiographies de contrôle sont indispensables pour s'assurer du caractère anatomique de la réduction, critère indispensable à un bon résultat fonctionnel. Le traitement doit être chirurgical en cas de luxation ouverte et d'irréductibilité. Si la réduction est stable, aucune ostéosynthèse n'est justifiée [2]. C'est le cas d notre patient. La rééducation reste la clé de tout bon résultat. Elle sera entreprise dès l'ablation du plâtre et ne peut être utilisée que s'il n'existe pas d'implants métalliques d'ostéosynthèse après un traitement chirurgical. Le pronostic de cette luxation est relativement bon chez la plupart des auteurs si la réduction est réalisée dans les heures suivant l'accident [12]. Le risque à craindre par la majorité des auteurs est l'arthrose sous talienne puis vient secondairement la nécrose talienne [13].

## Conclusion

La luxation astragalo-scapho-calcanéenne est une lésion rare et grave. Le diagnostic est clinique, confirmé par un examen radiologique. Cette luxation doit être traitée en urgence affin d'éviter la nécrose de la peau sous tendu, suivi d'une contention plâtrée pendant 6 semaines et d'un contrôle radiologique pour voir l'exactitude de la réduction rechercher les fractures associées et exclure un diastasis entre la malléole interne et le talus, témoin d'une lésion du ligament deltoïdien. Le traitement chirurgical n'est indiqué qu'en cas de fractures intra-articulaires déplacées ou d'irréductibilité. Une surveillance doit être long terme et le pronostic est en meilleur si ces principes sont respectés.

## Conflits d’intérêts

L'auteur ne déclare aucun conflit d'intérêts.
